# Supplementary material for: IMP regulates Kuzbanian to control the timing of Notch signalling in Drosophila follicle cells
Source: Development. 2019 Jan 18;146(2):dev168963. doi: 10.1242/dev.168963 (PMC6361131; doi:10.1242/dev.168963)
Supplement: Supplementary information [file develop-146-168963-s1.pdf]

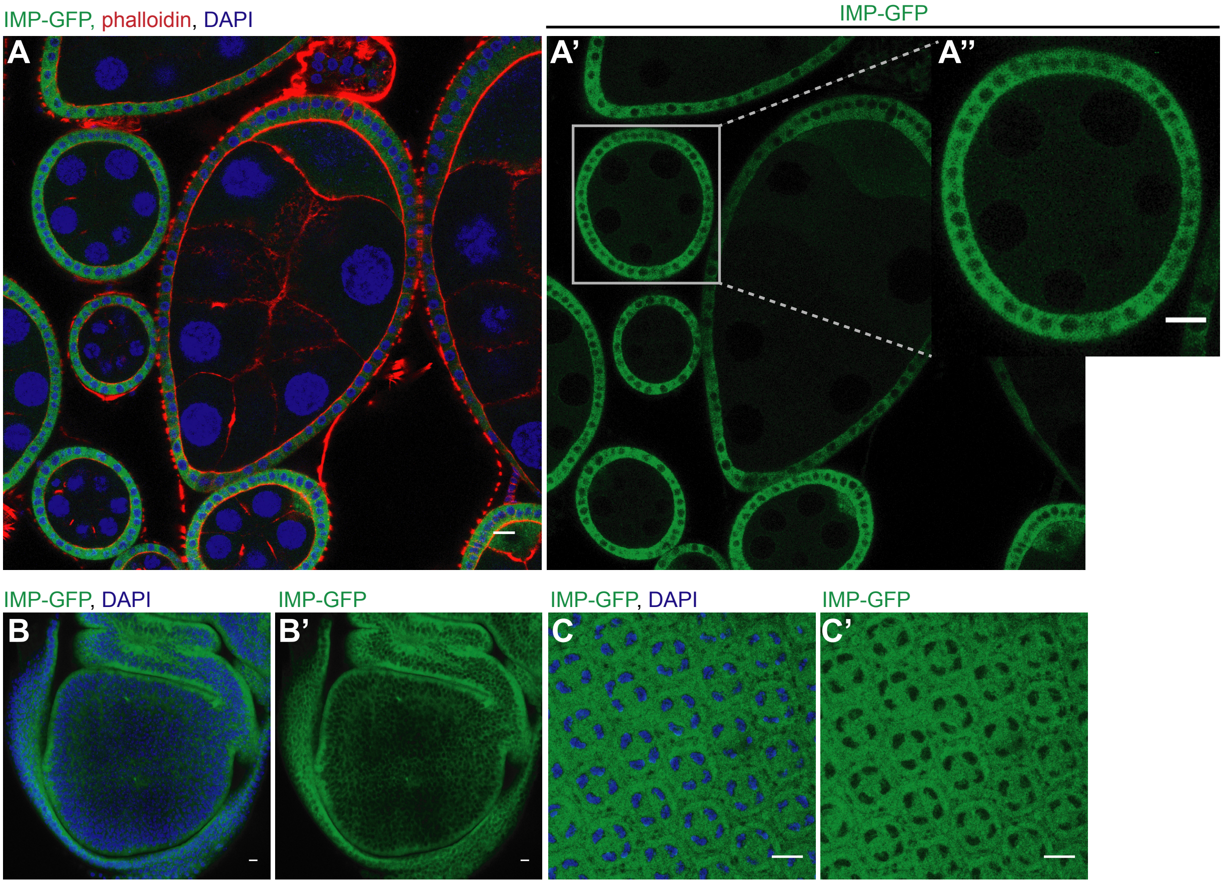

Fig. S1. IMP is uniformly distributed throughout the cytoplasm in different tissues.

(A-A") Wild-type egg chambers expressing IMP-GFP (green) from a protein trap insertion in the first intron, stained for actin (red) and DNA (blue). (A") shows the uniform distribution of IMP in the follicle cells at stage 6, when Dl/N signalling occurs.
(B-B') Z projection of confocal sections (10μm) through a wing imaginal disc expressing IMP-GFP (green) stained for DNA (blue). (B') IMP is evenly expressed throughout the wing pouch where Dl/N signalling is activated.
(C-C') Z projection of confocal sections (2μm) through a pupal eye imaginal disc expressing IMP-GFP (green) stained for DNA (blue). IMP is expressed in all cone cells. Scale bars: 10μm.

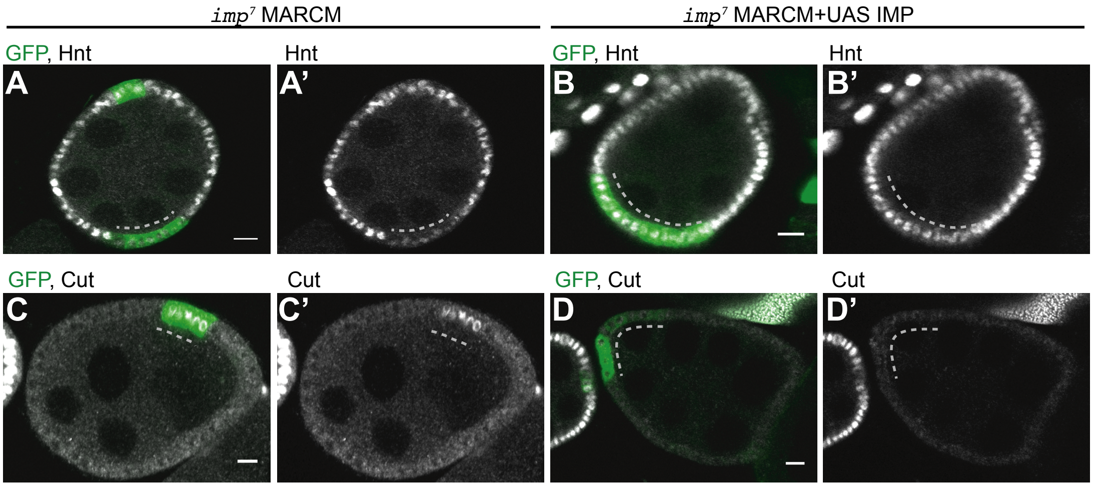

Fig. S2. Expression of UAS-IMP in *imp7* mutant MARCM clones rescues the delay in Notch signaling.

(A, C) Control *imp* mutant MARCM clones do not turn on Hnt (A) and do not downregulate Cut expression (C) at stage 7.
(B, D) Expression of UAS-IMP in the *imp* mutant cells restores timely Hnt expression (B) and Cut repression (D) at stage 7. Scale bars: 10μm.

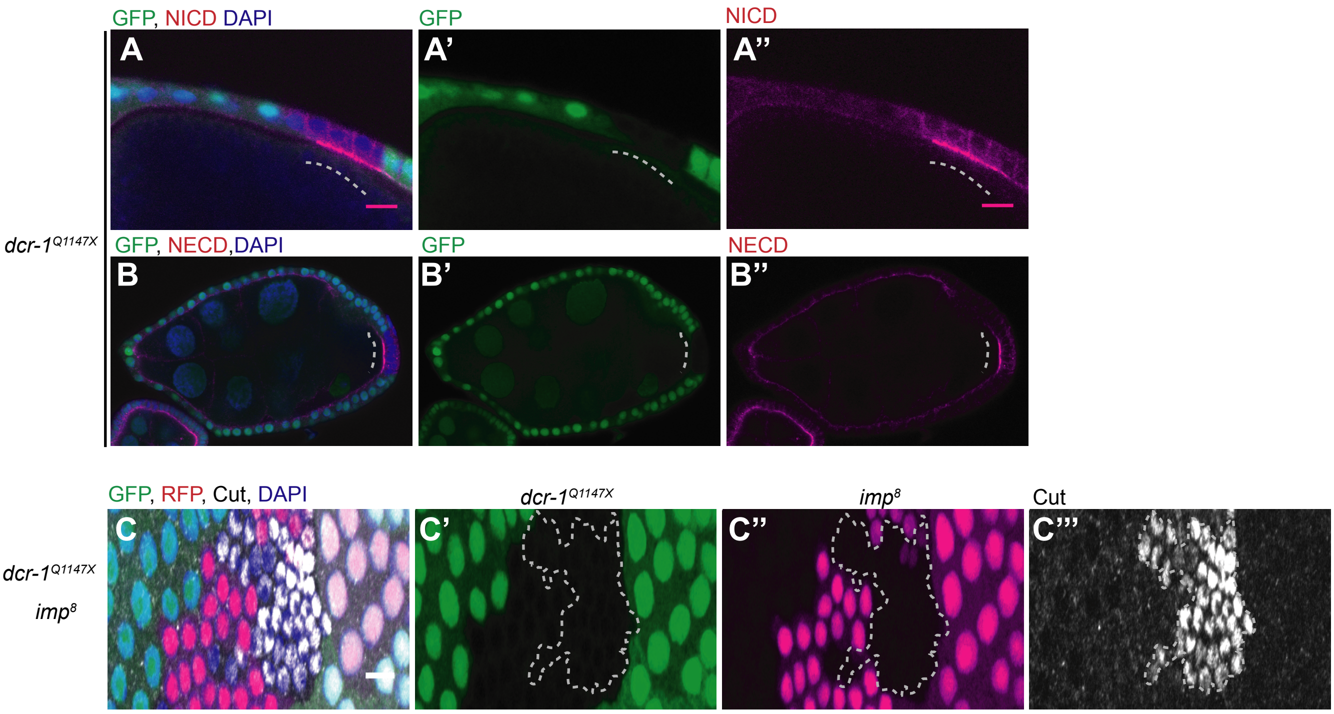

Fig. S3. IMP does not act through the micro RNA pathway.

(A-B) Stage 7 egg chambers containing dcr-1^{Q1147X} mutant clones marked by the loss of GFP (green). The mutant cells retain high levels of NICD (magenta in A, A', A") and NECD (magenta in B, B', B") at the apical membrane.

(C- C"') A stage 9 egg chamber containing both imp^{8} mutant clones marked by the loss of RFP (magenta) and dcr-1 mutant clones marked by the loss of GFP (green), stained for Cut (white). Cut is still expressed in the double mutant cells (surrounded by the dashed line), but not in the single mutant cells, indicating that loss of Dicer and IMP has an additive effect on the delay to Notch signalling. Scale bars: 10µm.

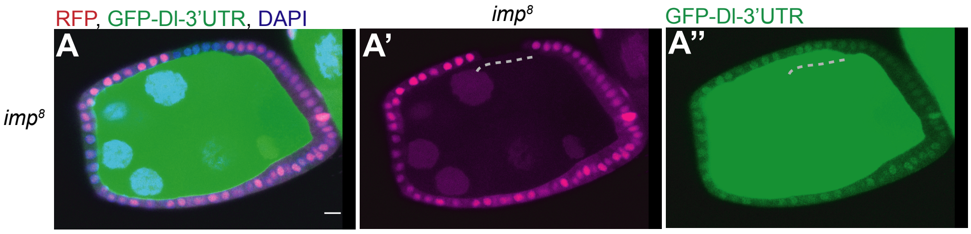

Fig. S4. IMP does not control the expression of a GFP-Dl 3'UTR sensor.

(A, A', A'') *imp*^{8} mutant cells marked by the loss of RFP (magenta) express the same levels of GFP from the GFP-Dl-3'UTR sensor as wild type cells. Scale bars: 10μm.

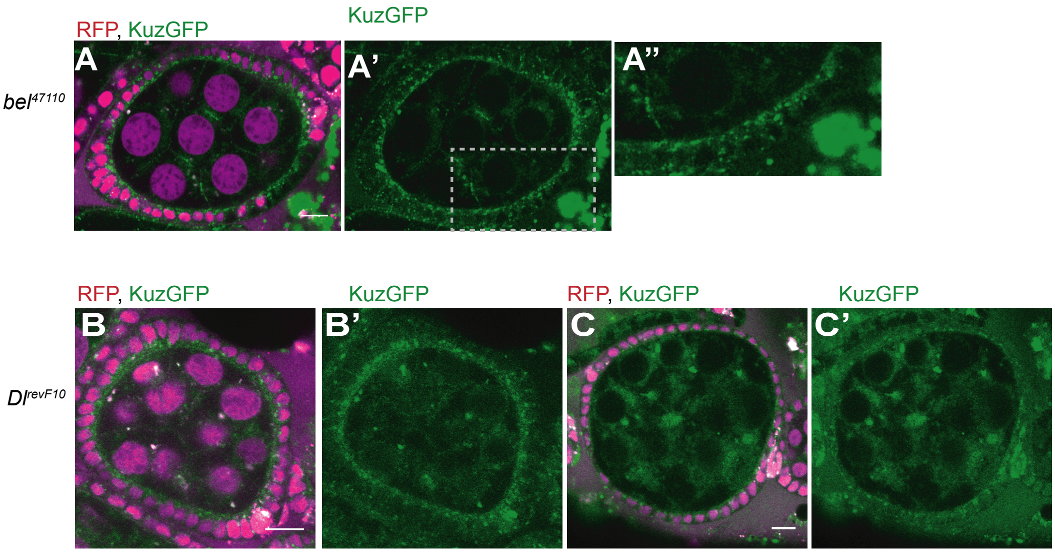

Fig. S5. Kuz-GFP foci form specifically in imp mutant cells and are not observed in *belle* mutant follicle cell clones or in follicle cells adjacent to *Delta* germline clones.

(A-A'') Kuz-GFP shows the same expression and localization in *bel* mutant and wild-type follicel cells. (B-C) Kuzbanian-GFP levels are reduced in the follicle cells surrounding *Delta* mutant germline cysts,but Kuz-GFP does not accumulate in endosomal foci. Scale bars: 10μm.

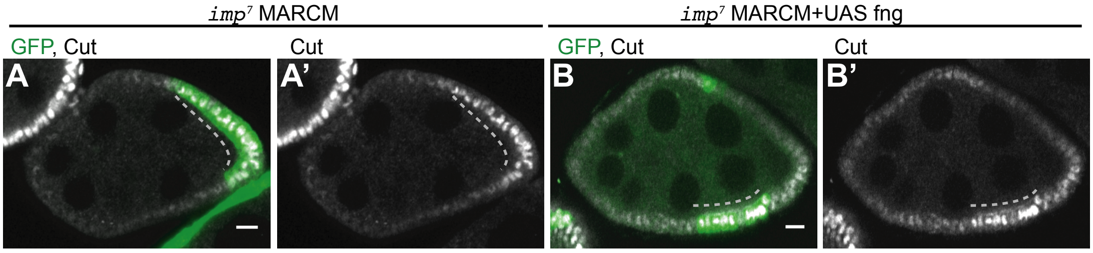

Fig. S6. Expression of UAS-fng in *imp7* mutant MARCM clones does not rescue the delay in Notch signaling.

(A, B) Stage 7 egg chambers containing *imp7 MARCM* clones marked by the expression of GFP (green) and stained for Cut (white). The continued expression of Cut in mutant cells at stage 7 is not rescued by the expression of Fng (C). Scale bars: 10µm.
